# The Effects of Subchronic Exposure to Metribuzin on *Danio rerio*

**DOI:** 10.1100/2012/728189

**Published:** 2012-06-18

**Authors:** Lucie Plhalova, Stanislava Stepanova, Eva Praskova, Lucie Chromcova, Lenka Zelnickova, Lenka Divisova, Misa Skoric, Vladimira Pistekova, Iveta Bedanova, Zdenka Svobodova

**Affiliations:** ^1^Department of Veterinary Public Health and Toxicology, Faculty of Veterinary Hygiene and Ecology, University of Veterinary and Pharmaceutical Sciences Brno, Palackeho 1/3, 612 42 Brno, Czech Republic; ^2^Department of Pathological Morphology, Faculty of Veterinary Medicine, University of Veterinary and Pharmaceutical Sciences Brno, Czech Republic

## Abstract

The aim of this study was to assess the impact of metribuzin in surface waters on fish under experimental conditions. The effects of subchronic exposure to metribuzin on fish growth and the development of histopathological changes in selected organs (gill, kidney, liver) and on activity of some biochemical markers (CYP450, EROD) in *Danio rerio* were investigated during a 28-day toxicity test. Juvenile growth tests were performed on *D. rerio* according to OECD method number 215. Fish at an initial age of 30 days were exposed to a range of metribuzin concentrations (1.5, 5, 16, 33, and 53 mg L^−1^). Exposure to metribuzin at 53 mg L^−1^ was associated with increased mortality. Negative effects with regard to total body weight, length, and the inhibition of specific growth rate were induced at concentrations of 33 and 53 mg L^−1^. Histopathological examination revealed pathological lesions in the liver in pesticide-exposed fish only at the highest concentration of 53 mg L^−1^ of metribuzin. Based on the results of growth rate, biochemical markers (CYP450, EROD), and histopathological examination, the lowest observed effect concentration (LOEC) value was 33 mg L^−1^ and the no observed effect concentration (NOEC) value was 16 mg L^−1^.

## 1. Introduction

Triazine herbicides are divided into two groups—symmetrical triazines, such as simazine, atrazine, propazine, cyanazine, ametryn, prometryn, prometon, and terbutryn, and asymmetrical triazines or triazinones, such as metribuzin [1]. Triazinones form a small group of herbicides developed in the 1970s for the pre- and postemergence control of grasses and broad-leaved weeds [2].

Metribuzin, which was first registered in Canada in 1971, is intended for the control of grasses and broad-leaved weeds in soyabeans, potatoes, tomatoes, sugar cane, alfalfa, asparagus, maize, and cereals. Metribuzin (4-amino-6-tert-butyl-4,5-dihydro-3-methylthio-1,2,4-triazin-5-one) is an inhibitor of photosynthesis. It is absorbed mainly by the roots but also by the leaves and is translocated in the xylem. Metribuzin (relative molecular mass 214.3) has a high water solubility (1.05 g L^−1^ at 20°C). Aqueous photolysis of metribuzin is rapid with a half-life of <1 day, and this clearly contributes to the half-life of <7 days in natural pond water. The half-life of metribuzin in soil is 1.5–4.0 months, and it is strongly bound to soils with high organic matter content. Soil surface photolysis in natural light proceeds with a half-life of 14–25 days [2, 3].

Due to the extensive use of herbicides in agriculture and their persistence, such triazines are present in surface and ground waters and may affect nontarget organisms [4–8] there. In a study of the presence of herbicides in Brazil, metribuzin was the most frequently detected herbicide (with a maximum concentration of 0.351 *μ*g L^−1^ [9]). Metribuzin is highly toxic to freshwater macrophytes and algae [10]. Concentrations of metribuzin lower than those affecting invertebrates and fish, for which it is moderately toxic, cause the inhibition of aquatic plant growth [3]. Metribuzin is more toxic to aquatic plants than other pesticides such as atrazine, alachlor, or metolachlor [11].

In 2008, metribuzin was detected (in trace amounts) in 21% of samples of Czech surface waters; however, in 2009, it was detected in 85% of samples. From 2009, polar organic chemical integrative samplers (POCISs) providing more accurate information in the long-term monitoring of pesticides have been used in the Czech Republic. POCIS is a passive in situ sampling device, which integratively concentrates trace levels of complex mixtures of hydrophilic environmental contaminants, enables the determination of their time-weighted average water concentrations, and provides a method of estimating the potential exposure of aquatic organisms to the complex mixture of waterborne contaminants [12].

Many studies have dealt with the determination of acute toxic concentrations of triazine herbicides or the chronic effects of triazine herbicides on various fish species [13–17]. However, the influence of metribuzin on fish, especially the chronic effects of sublethal concentrations of metribuzin, has not been fully examined or studied.

The aim of this study was to assess the impact of subchronic metribuzin exposure on fish growth and the development of histopathological changes in selected organs (gill, kidney, liver) and on alterations in some biochemical markers (CYP450, EROD) in *Danio rerio *during a 28-day toxicity test.

## 2. Material and Methods

### 2.1. Experimental Fish

Tests of metribuzin toxicity were performed on *Danio rerio*, which is one of the model organisms most commonly used in toxicity tests [18–20]. Experimental procedures were in compliance with national legislation (Act number 246/1992 Coll., on the Protection of Animals Against Cruelty, as amended and Decree number 207/2004 Coll., on the Protection, Breeding and Use of Experimental Animals, as amended).

### 2.2. The Subchronic Toxicity Test

The tests were performed on *D. rerio* at the age of 30 days, according to OECD number 215 Fish, Juvenile Growth Test. Aqueous testing solutions were prepared from Sencor 70 WG with metribuzin as the active compound at a concentration of 700 g kg^−1^ (AgroBio Opava, s.r.o.). The fish were randomly distributed into 30 liter glass aquaria, 40 specimens per each. The experiment was conducted in a flow-through system, and the volume of test solutions was replaced twice a day. The fish were exposed to a range of metribuzin concentrations (1.5, 5, 16, 33, and 53 mg L^−1^—there were the actual concentrations) for 28 days. The control was the control group with dilution water only. Each test on a metribuzin-treated group was performed in duplicate. The average initial weight of fish used in the experiment was 0.016 ± 0.009 g. The fish were fed with dried *Artemia salina* without nutshells to the amount of 8% of their body weight per day. The food ration was based on initial fish weights and was recalculated after 14 days. At the end of the tests, fish were weighed and their tank-average specific growth rates determined. Food was withheld from the fish 24 h prior to weighing.

During the tests, living conditions were checked at 24-hour intervals and the number of dead fish was recorded in each concentration. The mean values for water quality were temperature 25 ± 1°C, oxygen saturation above 60% (ranging from 76% to 95%), pH from 8.12 to 8.41. The basic chemical parameters of dilution water used were COD_Mn_ (chemical oxygen demand) 1.1–1.3 mg L^−1^; total ammonia below the limit of determination (<0.04 mg L^−1^); NO_3_^−^ 14.7–18.7 mg L^−1^; NO_2_^−^ below the limit of determination (<0.02 mg L^−1^); Cl^−^14.9–15.6 mg L^−1^; ∑ Ca ± Mg 11.5 mmol L^−1^.

Tank-average specific growth rates were calculated using the following formula according to OECD number 215:

(1) $$r=\frac{\bar{\text{lo}{\text{g}}_{e}W_{2}}-\bar{\text{lo}{\text{g}}_{e}W_{1}}}{t_{2}-t_{1}}*100,$$

*r* is tank-average specific growth rate, *W*_1_, *W*_2_ are weights of a particular fish at times *t*_1_ and *t*_2_, respectively, $\bar{\text{lo}{\text{g}}_{e}W_{1}}$ is average of the logarithms of the values *W*_1_ for the fish in the tank at the start of the study period, $\bar{\text{lo}{\text{g}}_{e}W_{2}}$ is average of the logarithms of the values *W*_2_ for the fish in the tank at the end of the study period, and *t*_1_, *t*_2_ are time (days) at the start and end of the study period.

### 2.3. Determination of Metribuzin

Gas chromatography with ion trap mass spectrometry (GC/IT-MS) was used for the determination of metribuzin concentrations. Sample preparation was based on simple liquid-liquid extraction into cyclohexane.

Separation, identification, and quantification of metribuzin were based on the GC/IT-MS method. A Varian 450-GC gas chromatograph (Varian Inc., USA) and VF-5 ms (30 m × 0.25 mm) column were used for the separation of metribuzin. A Varian 220-MS (Varian Inc., USA) ion trap mass spectrometer was used for identification and quantification. Chromatographic and MS conditions were based on methods described by Perreau and Einhorn [21]. All solvents were GC/MS-grade purity (Chromservis, s.r.o., CZ). Certified standard metribuzin was purchased from Dr. Ehrenstorfer GmbH (Germany).

The detection limit (3*σ*) of metribuzin was 0.05 *μ*g L^−1^. The expanded uncertainty of metribuzin was 7.2% on condition that the coefficient of expansion was *k* = 2.

### 2.4. Histopathological Examination

The fish were prepared for histopathological examination (of selected organs—gill, kidney, liver), fixed in buffered 10% neutral formalin, dehydrated, embedded in paraffin wax, sectioned on a microtome at a thickness of 4 *μ*m, and stained with haematoxylin and eosin (HE).

### 2.5. Biochemical Analyses

Whole body samples were homogenised in phosphate buffer (pH 7.4) and centrifuged (10,000 g, 20 min, 4°C), and the supernatant was recentrifuged (100,000 g, 1 h at 4°C). The final supernatant was drained, and the pellet was washed and resuspended in the phosphate buffer (pH 7.4). Each suspension in an Eppendorf tube was stored at −85°C until enzymatic assays. Microsomal protein concentrations were measured before the assays using the method described by Lowry et al. [22].

The total content of CYP450 in whole body samples was determined by visible light spectrophotometry (400 to 490 nm). Measurements were made following cytochrome reduction by sodium dithionite, after the complex with carbon monoxide was formed [23].

The catalytic activity concentration of EROD in whole body samples was measured by spectrofluorometry (excitation: 535 nm, emission: 585 nm). In the presence of the enzyme, the substrate 7-ethoxyresorufin is transformed into resorufin in the presence of nicotinamide adenine dinucleotide phosphate [23].

### 2.6. Statistical Analysis

Data were subjected to Kruskal-Wallis one-way ANOVA and subsequently to Dunnett's test in order to assess the statistical significance of differences in tank-average fish specific growth rates between test groups with different concentrations and those of the control groups. Estimation of the LOEC and NOEC was based on ANOVA followed by Dunnett's test for the identification of the lowest concentration at which these differences in specific growth rate and biochemical markers were (were not) significant at a 0.05 probability level and, further, on the results of histopathological examination and the assessment of changes in fish behaviour.

## 3. Results

### 3.1. Mortality and Fish Behaviour

**Figure 1:**
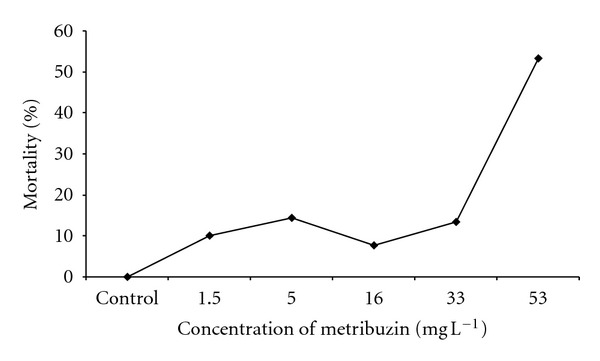
The mortality of *Danio rerio *at particular concentrations of metribuzin during the growth test (concentrations of metribuzin from 1.5 to 53 mg L^−1^).

In the control group, no mortality in fish was observed during the 28 day experimental period. Whereas the mortality in test groups at metribuzin concentrations of 1.5, 5, 16, and 33 mg L^−1^ was between 8 and 14%, the mortality at the highest concentration of metribuzin (53 mg L^−1^) even reached 53% (Figure 1). At concentrations of 33 and 53 mg L^−1^ of metribuzin, we also noticed decreased food intake compared to the control.

### 3.2. Growth Rate

**Figure 2:**
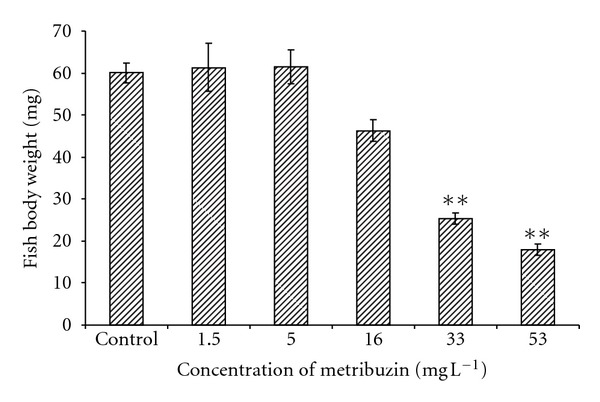
Comparison of body weight for control and tested concentrations at the end of the test (concentrations of metribuzin from 1.5 to 53 mg L^−1^) (**P < 0.01).

**Figure 3:**
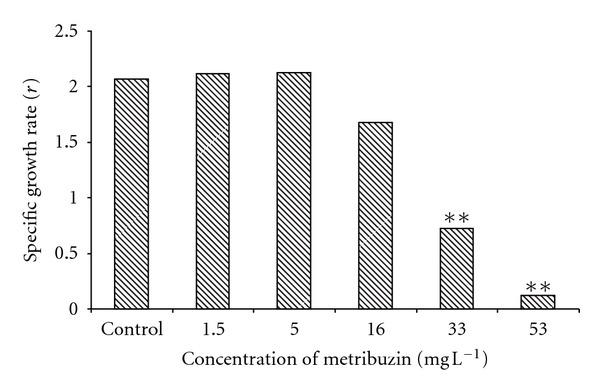
Comparison of specific growth rate (*r*) for control and tested metribuzin concentrations (concentrations of metribuzin from 1.5 to 53 mg L^−1^) (**P < 0.01).

The initial body weights were not significantly different between groups, but, at the end of the trial, body weights in tanks with 33 and 53 mg L^−1^ concentrations of metribuzin were significantly lower (*P* < 0.01) compared to the control group (mean ± SEM) (Figure 2). The values of specific growth rate *r* for the test groups in comparison with the control group are shown in Figure 3. A significant decrease (*P* < 0.01) in fish growth caused by metribuzin was seen at concentrations of 33 and 53 mg L^−1^.

We also found a significant decrease (*P* < 0.01) in individual fish total body length caused by metribuzin, which was detected at concentrations of 33 and 53 mg L^−1^.

### 3.3. Histopathological Examination

**Figure 4:**
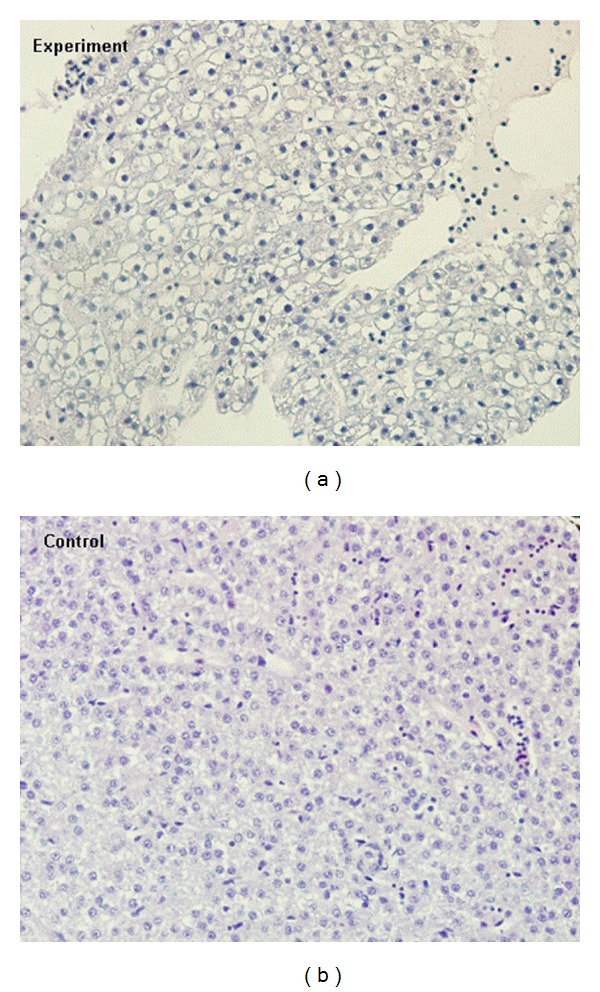
Diffuse hydropic to vacuolar degeneration of hepatocytes in the liver of *Danio rerio* exposed to metribuzin at a concentration of 53 mg L^−1^ for 28 days (HE, 600x).

Histopathological examination revealed pathological lesions in pesticide-exposed fish only in the experimental group with the highest concentration of 53 mg L^−1^ of metribuzin. Morphological changes were observed in the liver and represented by moderate dystrophic lesions of hepatocytes. There were morphological signs of initial cell injury represented by diffuse hydropic to vacuolar degeneration of hepatocytes (Figure 4). Affected tissues were histopathologically compared with tissue sections from the negative control group. Tissues and organs of the fish in experimental groups exposed to metribuzin at concentrations of 1.5, 5, 16, and 33 mg L^−1^ exhibited no pathomorphological changes.

### 3.4. Cytochrome P450 and EROD

There were no significant (*P* > 0.05) differences from controls in the concentration of total CYP and EROD in any treated group (1.5, 5, 16, 33, and 53 mg L^−1^ of metribuzin).

## 4. Discussion

Metribuzin is moderately toxic to aquaticinvertebrates and vertebrates [3]. Reported metribuzin 96hLC50 for various fish species ranged between 42 mg L^−1^ (*Oncorhynchus mykiss*) and 140 mg L^−1^for channel catfish [24–26]. For common carp (*Cyprinus carpio*), the metribuzin 96hLC50 was as much as 175.1 mg L^−1^ (250.2 mg L^−1^ of Sencor WG 70) [27].

On the basis of results of acute test for common carp determined by Velisek et al. [27], we chose for our test concentrations of metribuzin lower than LC50 (1.5, 5, 16, 33, and 53 mg L^−1^). Although the highest concentration of metribuzin in our tests (53 mg L^−1^) was less than one-third of the 96hLC50 for common carp, the mortality at this concentration was more than 50%. According to these results, the zebrafish appears to be more sensitive to metribuzin than common carp. The higher mortality at this concentration was accompanied by a reduction in the food intake of fish throughout the duration of the test (28 days).

Velisek et al. [27] described the behaviour of common carp at their metribuzin 96hLC50—fish were lying on the bottom of the tank and moving in circles, behaviour which was followed by a short excitation stage (convulsions). Velisek et al. [26] observed similar behaviour in acute test on rainbow trout (*Oncorhynchus mykiss*) (at 62.51 mg L^−1^ of metribuzin): accelerated respiration; loss of movement coordination; fish were lying on their flanks and moving in this position; the subsequent short excitation stage (convulsions, jumps above the water surface, movement in circles) changed into a resting stage, followed by another short-term excitation stage.

Fish in our test only swam in the middle of the tank with no signs of interest in food compared to control. We observed this change of behaviour at 33 mg L^−1^ and 53 mg L^−1^ of metribuzin, but, at 33 mg L^−1^ of metribuzin, mortality was not as high as at the highest concentration (only 13%). Behavioral changes have also been investigated after exposure to other triazines such as atrazine [14, 15]. According to Steinberg et al. [13], decreased food intake and other alterations in swimming behavior could be caused by the effect of atrazine on the sensory organs and nervous system.

Our results showed decreasing growth rates at 16 mg L^−1^ of metribuzin in comparison with the control group, but this decrease was not significant. However, we found statistically significant decreases in growth rates at concentrations of 33 and 53 mg L^−1^ of metribuzin. At other metribuzin concentrations (1.5 mg L^−1^ and 5 mg L^−1^) there were no significant differences from control in growth rates. Likewise, Modra et al. [28] did not notice any significant differences in body weight or hepatosomatic index in juvenile common carp at 1.75 mg L^−1^ of metribuzin after a 28-day exposure period. In addition, Fairchild and Sappington [10], who studied the impact of metribuzin on fish during a 56-day exposure period, did not find significant effects on the survival or growth of juvenile bluegill (*Lepomis macrochirus*) at 75 *μ*g L^−1^ of metribuzin.

Velisek et al. [26] observed histopathological alterations in the caudal kidneys and gills of rainbow trout after 96 h exposure to 89.3 mg L^−1^ of Sencor WG 70 (which corresponded to 62.51 mg L^−1^ of metribuzin)—the same pesticide preparation we used. They found hyaline degeneration in epithelial cells of the renal tubules and mild proliferation of goblet cells of the respiratory epithelium of secondary gill lamellae. The alteration of the kidney resulted in hypoproteinemia, followed by the formation of transudate in the body cavity. Velisek et al. [27] described the same histopathological changes in the kidney in common carp. We did not find any morphological changes in the gills and kidneys in all treated groups. Histopathological changes were observed only in the liver at the highest metribuzin concentration (53 mg L^−1^).

Similar histopathological changes have also been found after exposure to other triazines. Velisek et al. [29] reported changes in the histology of the liver (diffused steatosis with the loss of cellular shape and the presence of lipid inclusions in hepatic cells) and caudal kidney (destruction of renal tubules) in common carp after 28-day exposure to 40 *μ*g L^−1^ of terbutryn. Arufe et al. [30] detected pathological changes in the livers of yolk sac larvae of gilthead seabream (*Sparus aurata*) after 72 hours exposure to a commercial formulation containing simazine (4.5 mg L^−1^). Likewise, Oulmi et al. [31] and Fisher-Scherl et al. [32] described alterations to the kidneys in fish as a result of exposure to low concentrations of atrazine for 4 weeks.

In our study, the concentration of CYP and EROD activity did not increase. Likewise, Modra et al. [28], who exposed juvenile common carp to metribuzin at concentrations of 0.175 mg L^−1^ and 1.75 mg L^−1^ for 28 days, did not find increased activity of CYP450 or EROD. According to them, total cytochrome P450 concentration in fish is not a suitable biomarker of metribuzin pollution of the aquatic environment, because the amount of total CYP may not be affected—some xenobiotic compounds can act as inducers of specific isoenzymes but inhibit others [33, 34].

Based on our results concerning growth rate and histopathological examination, we estimated the LOEC (lowest observed effect concentration) and NOEC (no observed effect concentration) of metribuzin to be 33 mg L^−1^ and 16 mg L^−1^, respectively.

## Abbreviations

CYP450: Cytochrome P450
EROD: Ethoxyresorufin-*O*-deethylase
GC/IT-MS: Gas chromatography with ion trap mass spectrometry
NOEC: No observed effect concentration
LC50: 50% lethal concentration
LOEC: Lowest observed effect concentration
OECD: Organization for Economic Cooperation and Development
POCIS: Polar organic chemical integrative sampler.
